# Neural Correlates of Masking Level Differences: Evidence From Auditory Brainstem Responses

**DOI:** 10.1002/brb3.70725

**Published:** 2025-08-04

**Authors:** Aysenur Aykul Yagcioglu, Aysegul Esdogan, Aysun Parlak Kocabay

**Affiliations:** ^1^ Department of Audiology KTO Karatay University Konya Turkey; ^2^ Institute of Health Sciences, Doctorate Program of Audiology Hacettepe University Ankara Turkey; ^3^ Department of Audiology Hacettepe University Ankara Turkey

**Keywords:** auditory brainstem response, binaural integration, brainstem processing, masking level difference

## Abstract

**Objective:**

This study aimed to investigate the neural correlates of masking level differences (MLD) by examining their relationship with auditory brainstem responses (ABR) in normal‐hearing adults, to better elucidate the underlying brainstem mechanisms involved in MLD.

**Methods:**

A total of 37 normal‐hearing adults aged 18–30 years participated. Auditory evaluations included pure‐tone audiometry, tympanometry, MLD testing at 500 Hz under S₀N₀ and SπN₀ conditions, and click‐evoked ABR recordings. Correlations between ABR wave latencies (Waves I, III, and V) and interpeak intervals (I–III, III–V, and I–V) with MLD scores were analyzed using Spearman's correlation. Multiple linear regression analyses were conducted in two separate models to evaluate the predictive contribution of ABR components to MLD performance.

**Results:**

The mean MLD was 13.24 ± 2.18 dB, exceeding the clinical threshold for normal performance. No significant gender differences were observed in ABR or MLD measures. A moderate positive correlation was found between ABR Wave V latency and MLD scores (*r* = 0.58, *p* < 0.001). In regression analyses, Wave V latency was the only significant predictor of MLD scores (*p* = 0.003), explaining 28% of the variance. Other ABR components were not significant contributors.

**Conclusions:**

The results demonstrate that MLD performance is associated with ABR Wave V latency, suggesting that midbrain structures such as the inferior colliculus play a crucial role in binaural auditory processing. These findings support the utility of MLD as a behavioral index of brainstem integrity and its potential role in the evaluation of auditory dysfunction. While further research is needed to confirm these associations in clinical populations, MLD testing may offer valuable diagnostic insights into auditory brainstem dysfunction.

## Graphical abstract

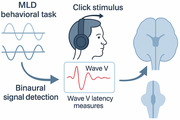

## 1 Introduction

Central auditory processing encompasses the mechanisms and processes involved in interpreting sound from its reception by the external ear to its perception in the auditory cortex. These skills include sound localization and lateralization, discrimination, and recognition of acoustic differences; temporal processing (resolution, masking, integration, and sequencing); and target stimulus perception (Back et al. 2021). Binaural interaction, dichotic listening, and temporal processing are critical components of auditory processing.

Binaural interaction refers to integrating and processing disparate but complementary information from both ears, beginning at the brainstem level. The superior olivary complex (SOC) is the first central auditory structure receiving inputs from both the ipsilateral and contralateral sides (Santiago et al. 2020). Behavioral tests assessing binaural integration typically present intensity, time, or spectral differences sequentially or simultaneously (ASHA 2025). One such test is the Masking Level Difference (MLD) test, which evaluates binaural integration at the brainstem level (McCullagh and Bamiou 2014; Santiago et al. 2020).

MLD is a measure of the superiority of the binaural hearing system and is an important tool for evaluating the complex processes of the auditory system (Goldstein and Stephens 1975). The MLD test is a psychoacoustic measure of the auditory system's sensitivity to differences in timing and amplitude between signals and noise. It differentiates between conditions in which the phase of the signal or the noise is manipulated. In the S0N0 condition, both signal and noise are presented in the same phase (homophasic), whereas in the SπN0 condition, the signal's phase is inverted by 180°, creating an antiphasic condition. The MLD is calculated by subtracting the threshold of the SπN0 condition from the S0N0 condition (McPherson et al. 2011). When the neurophysiological basis of MLD is examined, the auditory brainstem and cortex play a role in this process. It is stated that the timing of the signals required for the formation of MLD is encoded in the brainstem. This reveals the importance of central auditory processes in understanding the neurophysiological basis of MLD (Fowler 2017). MLD is particularly sensitive to brainstem lesions, especially those involving the SOC (McCullagh and Bamiou 2014). A reduced MLD value (< 7 dB) is often indicative of brainstem pathology (Brown and Musiek 2013; Gelfand 2016).

ABRs are evoked potentials recorded within 0–10 ms following an auditory stimulus. They reflect synchronous neural activity from the auditory nerve through the cochlear nucleus, SOC, lateral lemniscus, and inferior colliculus. Since both ABR and MLD originate at the brainstem level, examining their relationship may yield valuable insights into the neural mechanisms underlying MLD generation. Previous studies have explored the relationship between MLD and ABR, particularly in clinical or restricted samples. Jerger et al. (1982) examined patients with multiple sclerosis and reported that abnormalities in ABR Wave III were associated with reduced MLD scores, suggesting brainstem involvement in binaural integration (Jerger et al. 1982). Santiago et al. (2020) found correlations between MLD and both ABR and frequency‐following response (FFR) measures in a sample of young adult females, though their study did not examine interpeak latencies and lacked gender diversity (Santiago et al. 2020). Similarly, Hall and Grose (1993) demonstrated that children with a history of otitis media with effusion exhibited significantly lower MLD and prolonged ABR latencies, implying that early auditory experiences may affect brainstem processing (Hall and Grose 1993). In contrast, Kevanishvili and Lagidze (1987) recorded ABR, middle latency responses (MLR), and slow cortical potentials (SCP), concluding that MLD‐related effects were primarily reflected at the cortical rather than brainstem level (Kevanishvili and Lagidze 1987). Fowler (2017) provided a comprehensive review of MLD's electrophysiological basis and highlighted the need for additional empirical studies to clarify the neural mechanisms underlying binaural auditory processing (Fowler 2017). Taken together, these studies highlight the potential neural mechanisms underlying MLD but differ in methodology, participant characteristics, and scope of ABR analysis. The present study builds upon this literature by examining a sex‐balanced sample of normal‐hearing young adults and systematically analyzing multiple ABR components—including Waves I, III, V, and interpeak intervals—in direct relation to MLD scores. This approach aims to clarify the brainstem correlates of binaural integration through the combined use of behavioral and electrophysiological measures.

## 2 Materials and Method

### 2.1 Participants

All participants provided written informed consent before participation. The study was conducted in accordance with the principles outlined in the Declaration of Helsinki. The study was approved by the Local Ethical Committee (2025/008).

Sample size estimation was performed using G*Power 3.1 software. A minimum of 37 participants was required to achieve sufficient statistical power for correlation analyses, based on the parameters: two‐tailed test, expected correlation p(H1) = 0.5, *α* = 0.05, and power (1 − β) = 0.90. The study included 37 young adults (20 females, 17 males) aged between 18 and 30 years (mean 22.68 ± 3.06), with normal hearing thresholds (< 15 dB HL). Exclusion criteria included any history of otologic infections, otologic surgery, or neurological disorders.

### 2.2 Audiological Assessment

Pure‐tone audiometry was performed using an Interacoustics AC‐40 clinical audiometer. Air conduction thresholds were measured using TDH‐39 supra‐aural headphones, while bone conduction thresholds were assessed with a B‐81 bone vibrator. Hearing thresholds were determined across frequencies from 250 to 8000 Hz, following the Modified Hughson‐Westlake procedure. Tympanometric measurements were conducted using the Interacoustics Titan device with a 226 Hz probe tone at 85 dB SPL. Only individuals exhibiting Type A tympanograms, based on the Jerger classification, were included.

### 2.3 ABR Measurements

ABR recordings were obtained using the Socrates (Hedera Biomedics) system. Click stimuli were presented at 80 dB nHL at a rate of 11.7 stimuli per second with alternating polarity. Each recording consisted of 2000 sweeps. The bandpass filter was set between 30 and 3000 Hz. Absolute latencies of Waves I, III, and V, as well as interpeak latencies (I–III, III–V, and I–V), were measured. ABR measurements were taken separately for the right and left ears. In the analyses, in order to eliminate the difference between the ears and to balance the small variations in individual auditory transmission, the arithmetic average of the right and left ear data was taken for each wave and interpeak latency value, and this average value was used in the analyses.

### 2.4 MLD Assessment

The MLD test was performed using the integrated test module of the Interacoustics AC40 audiometer. In the test, a 500 Hz pure tone signal and a fixed wide‐band noise (masker) were presented binaurally (simultaneously to both ears). The masker level was kept constant at 50 dB HL. The two‐phase conditions used in the test are as follows. Homophasic condition (S₀N₀): The signal and masker were presented in the same phase in both ears. Antiphasic condition (SπN₀): The signal was presented with a 180° phase difference in one ear compared to the other, while the masker remained in the same phase in both ears. In both conditions, the initial level of the signal was set to 70 dB HL. The threshold determination process was carried out using the device's standard adaptive procedure. In this procedure, the signal level was reduced in 2 dB steps, and the lowest intensity level at which the participant correctly perceived the sound twice in a row was accepted as the threshold. Thresholds were determined separately in homophasic (S₀N₀) and antiphasic (SπN₀) conditions. The MLD value was calculated as the threshold difference between these two conditions:

$$\mathrm{MLD}=\mathrm{Threshold}\_S_{0}N_{0}-\mathrm{Threshold}\_S\pi N_{0}.$$

Since the test was conducted under binaural conditions, both ears were stimulated simultaneously. Using this method, binaural auditory processing capacity was evaluated.

### 2.5 Statistical Analysis

Data were analyzed using IBM SPSS Statistics for Windows, Version 25.0 (IBM Corp., Armonk, NY, USA). The normality of data distribution was assessed using the Shapiro–Wilk test, and the homogeneity of variances was evaluated with Levene's test. Latency values of ABR waves (I, III, V) and interpeak intervals (I–III, III–V, I–V) were compared between the right and left ears using a paired‐sample *t*‐test, as the data met the assumptions of normality. For the comparison of MLD values between male and female participants, the data did not meet parametric assumptions; therefore, the nonparametric Mann–Whitney *U* test was applied. To examine whether ABR wave latencies differed by gender, independent samples *t*‐tests were used, as these variables were normally distributed.

To investigate the association between MLD scores and auditory brainstem responses, bivariate correlations were first calculated using Spearman's rho, given that some variables violated normality assumptions. Bonferroni correction was applied to account for multiple comparisons across the six correlation tests between ABR components and MLD scores.

Subsequently, a multiple linear regression analysis was performed to determine which ABR parameters significantly predicted MLD scores. The MLD scores were evaluated as the dependent variable, and wave I, III, and V latency means and interpeak latency times (I–III, I–V, III–V) were evaluated as independent variables. Since a high level of correlation was observed between these variables, separate regression models were created for each variable group to reduce the risk of multicollinearity. In the first model, only wave I, III, and V means were used as independent variables; in the second model, interpeak latency values (I–III, I–V, III–V) were included. The model fit was assessed using residual analyses and standard goodness‐of‐fit statistics. Collinearity diagnostics, including tolerance and variance inflation factor (VIF) values, were within acceptable limits, indicating no violation of the multicollinearity assumption. Examination of residual and scatter plots confirmed that the assumptions of normality, linearity, and homoscedasticity were satisfied. A *p* value of less than 0.05 was considered statistically significant.

## 3 Results

### 3.1 ABR Latencies between Ears

Table 1 presents the mean latencies of ABR Waves I, III, and V, along with interpeak intervals (I–III, III–V, and I–V) for the right and left ears. Latencies for Waves I, III, and V were significantly longer in the left ear compared to the right ear (all *p* values < 0.001).

**TABLE 1:** Latencies of ABR waves I, III, and V and interpeak intervals for the right and left ears.

ABR Parameter	Ear	N	Mean (ms)	*p*
Wave I	Right	37	1.54 ± 0.18	< 0.001
	Left	37	1.61 ± 0.17	
Wave III	Right	37	3.54 ± 0.21	< 0.001
	Left	37	3.66 ± 0.17	
Wave V	Right	37	5.46 ± 0.17	< 0.001
	Left	37	5.50 ± 0.19	
Interpeak I–III	Right	37	2.00 ± 0.23	< 0.001
	Left	37	2.05 ± 0.2	
Interpeak III–V	Right	37	1.93 ± 0.17	< 0.001
	Left	37	1.84 ± 0.18	
Interpeak I–V	Right	37	3.93 ± 0.23	< 0.001
	Left	37	3.90 ± 0.23	

### 3.2 ABR Latencies by Gender

**FIGURE 1:**
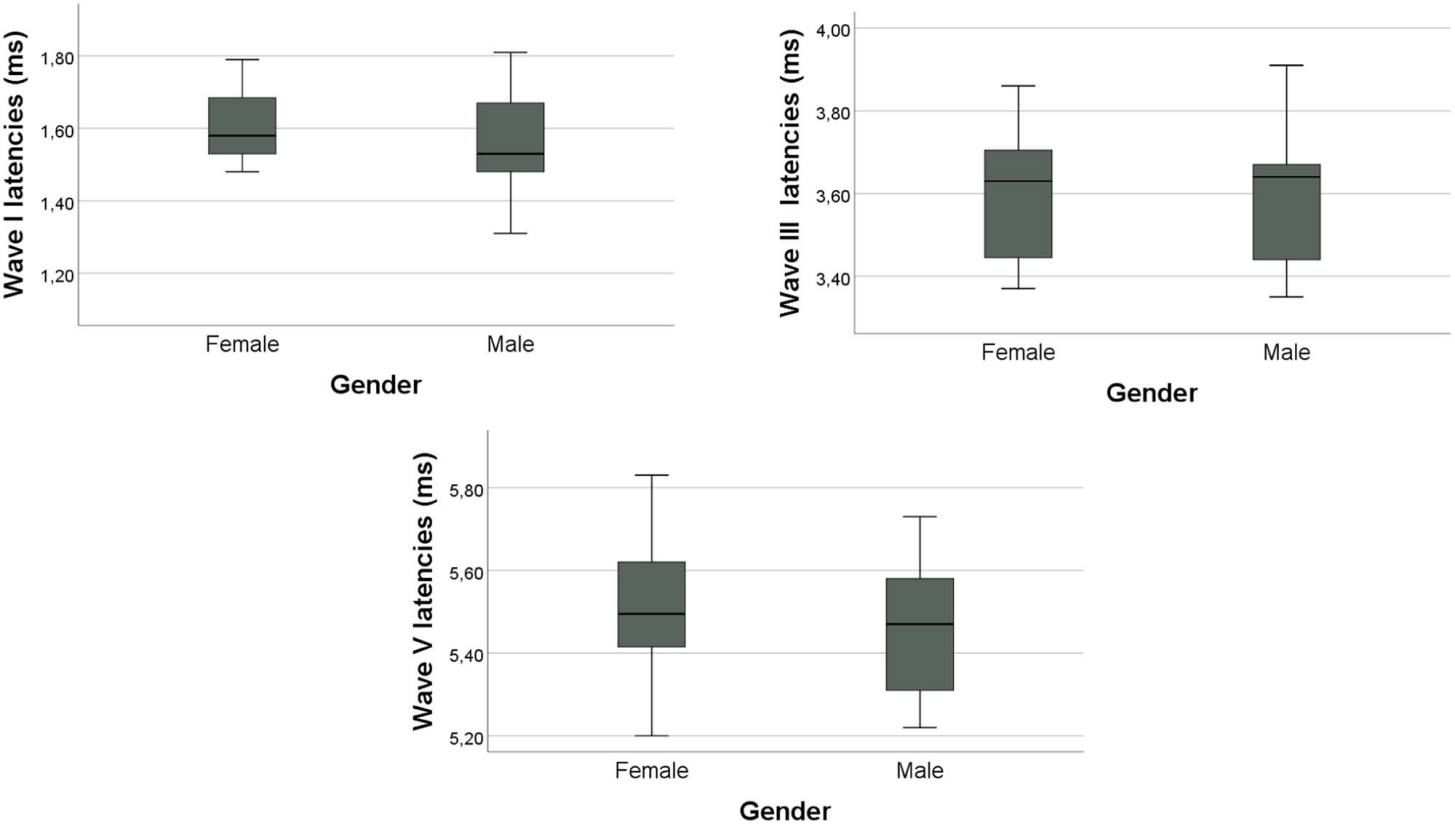
ABR wave latencies by gender.

No statistically significant gender differences were found for ABR Wave I, III, or V latencies (Figure 1). Specifically, the mean latency of Wave I was 1.59 ± 0.14 ms in females and 1.56 ± 0.15 ms in males (*p* = 0.53). Wave III latency was identical between genders (3.60 ± 0.15 ms for females and 3.60 ± 0.16 ms for males, *p* = 0.93). Similarly, Wave V latency showed no significant gender‐based difference (5.52 ± 0.17 ms in females vs. 5.46 ± 0.15 ms in males, *p* = 0.29).

### 3.3 MLD Performance by Gender

**FIGURE 2:**
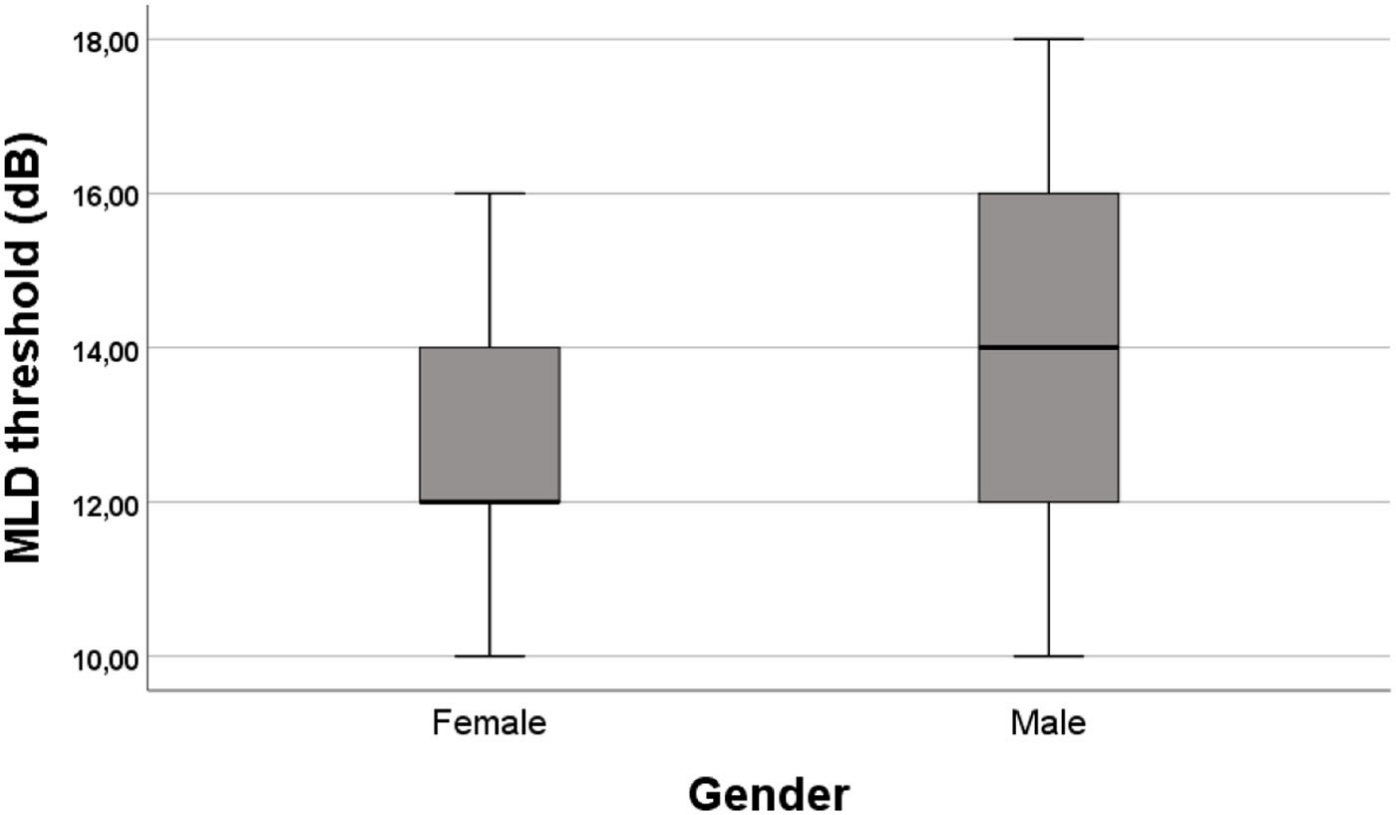
Mean MLD results by gender.

The MLD scores ranged from 10 to 18 dB, with a mean of 13.24 ± 2.18 dB across participants. Mean MLD values were 12.90 ± 2.10 dB for females and 13.65 ± 2.26 dB for males (Figure 2). No statistically significant gender differences were observed in MLD performance (*p* > 0.05).

### 3.4 Correlation Between ABR Latencies and MLD

**FIGURE 3:**
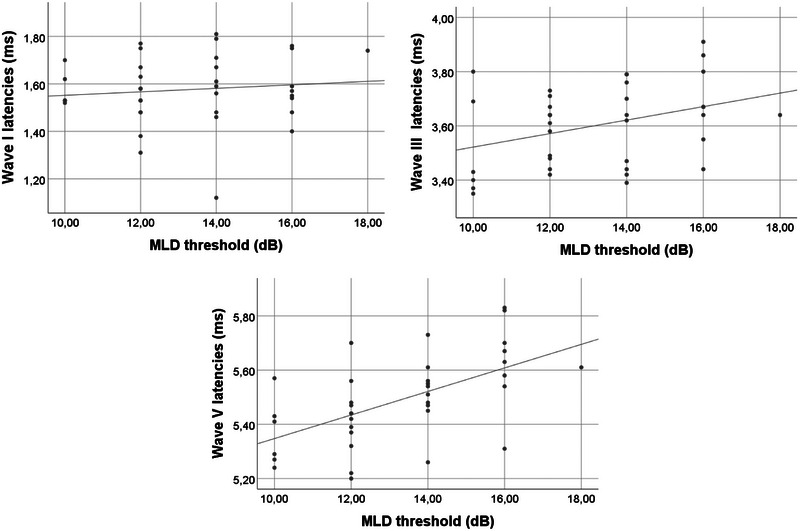
Correlations of ABR wave latencies with MLD.

Figure 3 and Table 2 summarize the correlations between ABR parameters and MLD scores. A moderate positive correlation was found between ABR Wave V latency and mean MLD (*r* = 0.61, *p* < 0.001). No significant correlations were observed for Wave I and Wave III latency, interpeak I–III, or interpeak III–V intervals. Bonferroni correction was applied for the six correlation tests, resulting in an adjusted significance threshold of *p* < 0.0083. Corrected and uncorrected *p* values are presented in Table 2.

**TABLE 2:** Correlations between ABR latencies/interpeak intervals and MLD

ABR parameter	Correlation with MLD (r)	*p*	Adjusted‐*p* value
Wave I	0.15	0.37	1.00
Wave III	0.33	0.04	0.24
Wave V	0.61	< 0.001	< 0.001*
Interpeak I–III	0.22	0.19	1.00
Interpeak III–V	0.32	0.05	0.30
Interpeak I–V	0.37	0.02	0.12

* indicates statistical significance of adjusted p values at < 0.0083, after Bonferroni correction.

### 3.5 Regression Analyses Predicting MLD Scores

To examine the predictive value of ABR parameters on MLD scores, two separate multiple linear regression models were constructed to avoid multicollinearity between wave and interpeak latency variables. In the first model, Wave I, III, and V latencies were entered as independent variables. In the second model, interpeak latency intervals (I–III, III–V, and I–V) were used.

The first model was statistically significant (*F* = 5.71, *p* = 0.003) and explained 28% of the variance in MLD scores (adjusted *R* ^2^ = 0.28). Among the predictors, only Wave V latency emerged as a significant predictor (*β* = 0.626, *p* = 0.003) whereas Wave I (*β* = 0.045, *p* = 0.758) and Wave III (*β* = −0.075, *p* = 0.702) latencies did not contribute significantly.

The second model, including interpeak latencies, was also statistically significant (*F* = 3.64, *p* = 0.037), but accounted for a smaller proportion of the variance in MLD scores (adjusted *R* ^2^ = 0.13). None of the individual predictors in this model reached statistical significance (all *p* > 0.05), indicating that although the overall model fit was acceptable, the interpeak intervals did not independently contribute to the prediction of MLD.

## 4 Discussion

The aim of this study was to investigate the neural correlates of MLD by examining its relationship with ABR parameters in normal‐hearing adults. The findings revealed a moderate positive correlation between MLD scores and ABR Wave V latency, supporting the involvement of midbrain structures in binaural auditory processing.

ABR and MLD are two key measures that provide complementary insights into the neural mechanisms of auditory brainstem processing. Although there have been studies investigating the neural generators of the ABR, the specific generator sites for most ABR waves are still unclear, except for the certainty that wave I is generated in the distal portion of the auditory nerve. It is generally accepted that ABR wave generators originate from the eighth cranial nerve and the brainstem region along the lateral lemniscus and inferior colliculus, but the specific generators of ABR waves are complicated by the fact that specific anatomical regions contribute to the generation of multiple waves (Krishnan 2023). The SOC is the first site in the auditory system to process binaural input. Although this structure was previously thought to be the source of wave III, studies have shown that the cochlear nucleus is the primary generator of wave III responses and that ascending structures of the auditory pathway that are more central than the cochlear nucleus are unlikely to contribute to wave III generation (Moller and Jannetta 1983). Responses recorded from the lateral brainstem close to the SOC showed that the SOC is the main generator of wave IV (Møller et al. 1994). MLD, on the other hand, reflects the auditory system's ability to separate target signals from background noise and is also known to originate at the brainstem level. Since both ABR and MLD are mediated by overlapping neural generators, examining their relationship may provide valuable insight into the physiological basis of binaural processing.

MLD is thought to originate primarily at the brainstem level, where auditory inputs from both ears converge on the SOC both ipsilaterally and contralaterally. Animal studies have provided robust evidence supporting this view. For example, experiments in protozoa, chinchillas, and guinea pigs have demonstrated that MLD reflects brainstem‐level processing (Mandava et al., 1996; Palmer et al. 1999; Wong and Stapells 2004). While ABR provides an objective electrophysiological index of auditory brainstem function, MLD complements this by behaviorally capturing binaural performance. However, despite sharing common neural generators, the relationship between these two tests has been systematically investigated in only a limited number of studies, and the findings remain inconsistent. (Jerger et al. 1982; Santiago et al. 2020; Hall and Grose 1993; Kevanishvili and Lagidze 1987).

Our findings that only Wave V latency is significantly associated with MLD scores, while other wave and interpeak latencies are not, likely reflect the unique neurophysiological role of midbrain structures in binaural integration. ABR Wave V is primarily generated at the level of the inferior colliculus, with contributions from the lateral lemniscus (Møller and Jannetta 1983; Krishnan 2023). These structures play a critical role in processing interaural phase and time differences—the same acoustic cues that underlie the MLD phenomenon. In contrast, Wave I originates from the auditory nerve and reflects peripheral conduction, while Wave III is associated with the cochlear nucleus and possibly the SOC. These earlier components likely play less direct roles in the higher‐order binaural integration needed for MLD performance. Similarly, interpeak intervals such as I–III and III–V did not significantly predict MLD scores because these intervals primarily reflect neural conduction from more peripheral structures rather than the central integrative processing required for binaural unmasking. The I–V interpeak interval spans the full brainstem, and it reflects a more generalized conduction time rather than the specific binaural integration required for MLD performance. Our findings are consistent with neuroanatomical models and auditory processing frameworks, which emphasize the role of the inferior colliculus in integrating binaural information (Palmer et al. 1999; Wong and Stapells 2004), supporting the conclusion that midbrain auditory synchrony plays a dominant role in predicting MLD outcomes.

Several previous studies have explored the relationship between MLD and ABR, but they differ in methodology, sample characteristics, and analytical scope. Jerger et al. (1982) investigated individuals with multiple sclerosis and found that abnormal Wave III latencies were associated with reduced MLDs, suggesting a brainstem origin, but their clinical sample limited generalizability. Hall and Grose (1993) showed that children with a history of otitis media exhibited prolonged ABR latencies (including Waves III and V) and reduced MLD scores, indicating early auditory experience may affect brainstem processing, though this study also focused on a clinical pediatric group. Kevanishvili and Lagidze (1987) reported no significant differences in ABR between MLD conditions (S₀N₀ vs. SπN₀), concluding that MLD might be cortical in origin; however, their conclusions are limited by the very small sample size (*n* = 6), and their findings conflict with more recent data. Santiago et al. (2020) assessed MLD and ABR in normal‐hearing adult females and found a correlation between Wave V latency and MLD. However, their study included only female participants and did not examine interpeak intervals. Fowler (2017) provided a theoretical synthesis of electrophysiological data on MLD but called for more empirical evidence linking specific ABR components to behavioral performance. Our study extends this body of work in several critical ways. First, unlike most prior studies that used either clinical populations or limited sample characteristics (e.g., only children or only females), we examined a sex‐balanced sample of normal‐hearing adults, enhancing generalizability. Second, while earlier research largely relied on correlation, we applied multiple linear regression models to independently evaluate the predictive value of both ABR wave and interpeak latencies for MLD performance. Notably, our results revealed that Wave V latency was a significant predictor of MLD scores, suggesting that midbrain structures such as the inferior colliculus play a dominant role in binaural integration. These findings clarify the physiological underpinnings of MLD performance and highlight the value of using combined ABR and MLD assessment to probe central auditory function in both research and clinical contexts.

Despite its strengths, the present study has limitations. The sample consisted exclusively of young adults with normal hearing, and the study evaluated only ABR and MLD tests; it did not include other central auditory tests or advanced neuroimaging methods. Future research should involve larger, more diverse samples, including clinical populations with confirmed brainstem lesions, to further evaluate the diagnostic utility of combining ABR and MLD assessments.

From a clinical perspective, integrating ABR and MLD testing may enhance diagnostic sensitivity in identifying brainstem pathologies. For example, in neurological conditions such as multiple sclerosis, where demyelination may disrupt both timing and synchrony within auditory pathways, MLD may serve as a sensitive behavioral marker of central auditory dysfunction (Fowler 2017; Wong and Stapells 2004). The dual use of these measures could aid in early detection, especially in cases where standard audiological tests are insufficient.

## 5 Conclusions

This study examined the relationships between MLD scores and ABR components and found that wave V latency in particular significantly predicted MLD scores. This finding suggests that mid‐level structures, especially centers such as the inferior colliculus, contribute more strongly to binaural auditory processing than early brainstem structures. The results suggest that the MLD test provides an indirect view of brainstem integrity as a behavioral measure and can be used as a complementary tool in the analysis of central auditory function when evaluated together with ABR data. In cases where functional disorders at the brainstem level are suspected, the inclusion of the MLD test in auditory evaluations may provide potential clinical benefit. However, further studies, including clinical populations, are needed to confirm these relationships and to evaluate the diagnostic validity of the test more comprehensively.

## Author Contributions

**Aysenur Aykul Yagcioglu**: conceptualization, methodology, investigation, data curation, formal analysis, visualization, writing – original draft. **Aysegul Esdogan**: investigation, data curation, writing – original draft. **Aysun Parlak Kocabay**: writing – original draft, writing – review and editing, formal analysis.

## Conflicts of Interest

The authors declare no conflicts of interest.

## Peer Review

The peer review history for this article is available at https://publons.com/publon/10.1002/brb3.70725

## Data Availability

The data that support the findings of this study are available on request from the corresponding author. The data are not publicly available due to privacy or ethical restrictions.
